# (4*S*)-5′-Chloro-3,7,7-trimethyl-5,6,7,8-tetra­hydro-4*H*-spiro­[1,2-oxazolo[5,4-*b*]quinoline-4,3′-indole]-2′,5-dione

**DOI:** 10.1107/S1600536814000191

**Published:** 2014-01-18

**Authors:** E. Govindan, PanneerSelvam Yuvaraj, Boreddy Siva Rami Reddy, K. Premalatha, A. SubbiahPandi

**Affiliations:** a Department of Physics, Presidency College (Autonomous), Chennai 600 005, India; b Industrial Chemistry Laboratory, Central Leather Research Institute, Adyar, Chennai 600 020, India; c D. G. Vaishnav College (Autonomous), Arumbakkam, Chennai 600 106, India

## Abstract

In the title compound, C_20_H_18_ClN_3_O_3_, the five- and six-membered heterocycles fused through a spiro C atom are inclined to each other at an angle of 87.4 (1)°. In the tricyclic ring system, the cyclo­hexene ring adopts an envelope conformation with the spiro atom as the flap. In the crystal, two sets of N—H⋯O hydrogen bonds link the mol­ecules into columns containing centrosymmetric *R*_2_^2^(7) ring motifs and propagating along the *b-*axis direction.

## Related literature   

For applications of indole, quinoline and pyrrolidine derivatives, see: Padwa *et al.* (1999 ▶). For hydrogen-bond motifs, see: Bernstein *et al.* (1995 ▶).
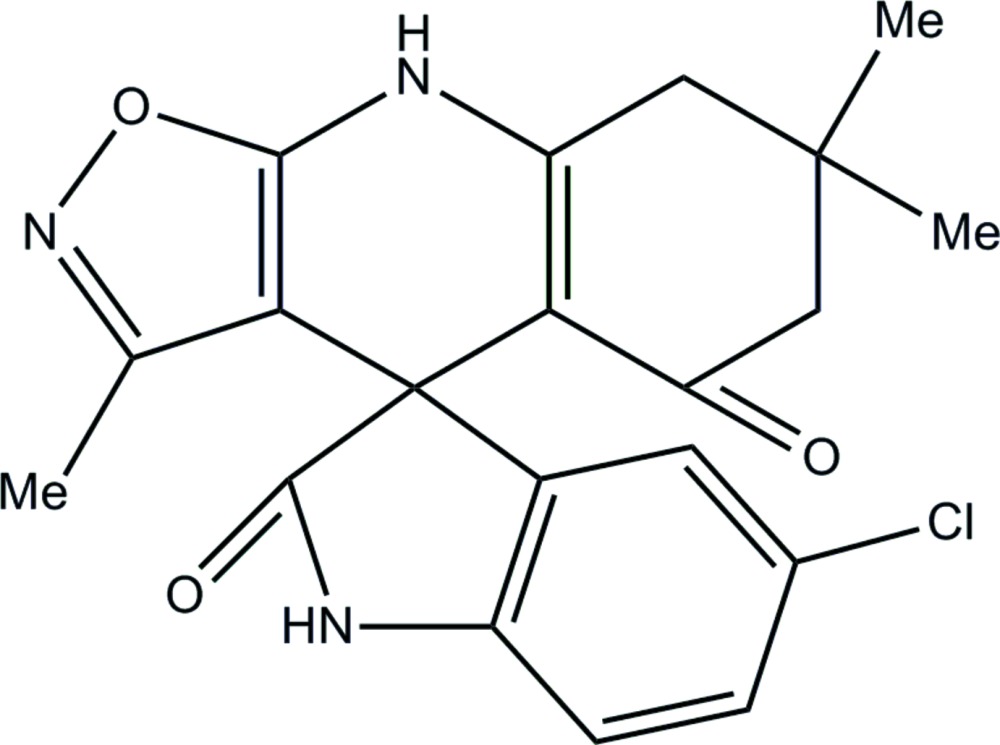


## Experimental   

### 

#### Crystal data  

C_20_H_18_ClN_3_O_3_
*M* *_r_* = 383.82
Orthorhombic, 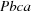

*a* = 17.9320 (7) Å
*b* = 11.1120 (4) Å
*c* = 18.5968 (7) Å
*V* = 3705.6 (2) Å^3^
*Z* = 8
Mo *K*α radiation
μ = 0.23 mm^−1^
*T* = 293 K
0.21 × 0.19 × 0.18 mm

#### Data collection  

Bruker SMART APEXII CCD diffractometer
Absorption correction: multi-scan (*SADABS*; Bruker, 2008 ▶) *T*_min_ = 0.952, *T*_max_ = 0.959
17841 measured reflections
4590 independent reflections
2643 reflections with *I* > 2σ(*I*)
*R*_int_ = 0.032

#### Refinement  

*R*[*F* ^2^ > 2σ(*F* ^2^)] = 0.044
*wR*(*F* ^2^) = 0.134
*S* = 1.01
4590 reflections
247 parameters
H-atom parameters constrained
Δρ_max_ = 0.29 e Å^−3^
Δρ_min_ = −0.36 e Å^−3^

### 

Data collection: *APEX2* (Bruker, 2008 ▶); cell refinement: *SAINT* (Bruker, 2008 ▶); data reduction: *SAINT*; program(s) used to solve structure: *SHELXS97* (Sheldrick, 2008 ▶); program(s) used to refine structure: *SHELXL97* (Sheldrick, 2008 ▶); molecular graphics: *ORTEP-3 for Windows* (Farrugia, 2012 ▶); software used to prepare material for publication: *SHELXL97* and *PLATON* (Spek, 2009 ▶).

## Supplementary Material

Crystal structure: contains datablock(s) global, I. DOI: 10.1107/S1600536814000191/cv5438sup1.cif

Structure factors: contains datablock(s) I. DOI: 10.1107/S1600536814000191/cv5438Isup2.hkl

Supporting information file. DOI: 10.1107/S1600536814000191/cv5438Isup3.cml

CCDC reference:

Additional supporting information:  crystallographic information; 3D view; checkCIF report

## Figures and Tables

**Table 1:** Hydrogen-bond geometry (Å, °)

*D*—H⋯*A*	*D*—H	H⋯*A*	*D*⋯*A*	*D*—H⋯*A*
N1—H1⋯O3^i^	0.86	2.05	2.837 (2)	151
N3—H3⋯O1^ii^	0.86	2.00	2.795 (2)	153

Symmetry codes: (i) 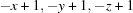; (ii) 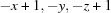.

**Fig. 1.**
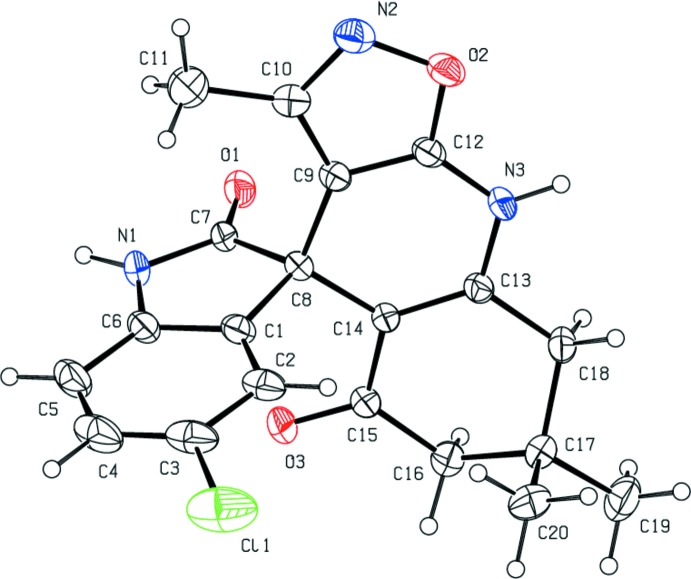
The molecular structure of the title compound, showing the atomic numbering and displacement ellipsoids drawn at 30% probability level.

## supplementary crystallographic information

### 1. Comment

A large number of natural products contain the quinoline and indole heterocycles, and they are found in numerous commercial products, including pharmaceuticals, fragrances and dyes (Padwa *et al.*, 1999). In view of the above importance, crystallographic study of the title compound (I) has been carried out to establish its molecular structure.

In (I) (Fig. 1), the indole ring adopts slightly envelope conformation on atom C7. The sum of the bond angle around atom N3 (360°) of the quinoline ring indicates *sp*^2^ hybridization. The quinoline group and indole ring are in axial orientation with the dihedral angle between them as 87.39 (1)°. The indole and quinoline ring systems are planar and keto atoms O1 and O3 deviate from the attached ring system by -0.024 (1) and -0.012 (2) Å, respectively.

In the crystal, molecules are linked by two sets of N—H···O hydrogen bonds (Table 1), forming centrosymmetric dimers containing two *R*^2^_2_(7) ring motifs (Bernstein *et al.*, 1995).

### 2. Experimental

A reaction mixture of 5-chloro isatin (1 mmol), 5,5-dimethylcyclohexane-1,3 dione (1 mmol) and 5-amino-3-methylisoxazole (1 mmol) in 5 ml of ethanol was heated up to 80°C for 6–10 h. The reaction was monitored by TLC. Then, the reaction mixture was filtered hot and the resulting solid products were washed with ethanol, dried in an air and recrystallized from ethanol.

### 3. Refinement

All H atoms were positioned geometrically (C–H = 0.93–0.98 Å, N–H = 0.86 Å) and allowed to ride on their parent atoms, with *U*_iso_(H) = 1.2–1.5*U*_eq_(C, N).

### Crystal data

**Table:**

C_20_H_18_ClN_3_O_3_	*F*(000) = 1600
*M**_r_* = 383.82	*D*_x_ = 1.376 Mg m^−^^3^
Orthorhombic, *P**b**c**a*	Mo *K*α radiation, λ = 0.71073 Å
Hall symbol: -P 2ac 2ab	Cell parameters from 2643 reflections
*a* = 17.9320 (7) Å	θ = 2.2–28.3°
*b* = 11.1120 (4) Å	µ = 0.23 mm^−^^1^
*c* = 18.5968 (7) Å	*T* = 293 K
*V* = 3705.6 (2) Å^3^	Block, white
*Z* = 8	0.21 × 0.19 × 0.18 mm

### Data collection

**Table:**

Bruker SMART APEXII CCD diffractometer	4590 independent reflections
Radiation source: fine-focus sealed tube	2643 reflections with *I* > 2σ(*I*)
Graphite monochromator	*R*_int_ = 0.032
ω and φ scans	θ_max_ = 28.3°, θ_min_ = 2.2°
Absorption correction: multi-scan (*SADABS*; Bruker, 2008)	*h* = −22→23
*T*_min_ = 0.952, *T*_max_ = 0.959	*k* = −14→12
17841 measured reflections	*l* = −24→24

### Refinement

**Table:**

Refinement on *F*^2^	Primary atom site location: structure-invariant direct methods
Least-squares matrix: full	Secondary atom site location: difference Fourier map
*R*[*F*^2^ > 2σ(*F*^2^)] = 0.044	Hydrogen site location: inferred from neighbouring sites
*wR*(*F*^2^) = 0.134	H-atom parameters constrained
*S* = 1.01	*w* = 1/[σ^2^(*F*_o_^2^) + (0.0522*P*)^2^ + 1.5319*P*] where *P* = (*F*_o_^2^ + 2*F*_c_^2^)/3
4590 reflections	(Δ/σ)_max_ < 0.001
247 parameters	Δρ_max_ = 0.29 e Å^−^^3^
0 restraints	Δρ_min_ = −0.36 e Å^−^^3^

### Special details

**Table:**

Geometry. All e.s.d.'s (except the e.s.d. in the dihedral angle between two l.s. planes) are estimated using the full covariance matrix. The cell e.s.d.'s are taken into account individually in the estimation of e.s.d.'s in distances, angles and torsion angles; correlations between e.s.d.'s in cell parameters are only used when they are defined by crystal symmetry. An approximate (isotropic) treatment of cell e.s.d.'s is used for estimating e.s.d.'s involving l.s. planes.
Refinement. Refinement of *F*^2^ against ALL reflections. The weighted *R*-factor *wR* and goodness of fit *S* are based on *F*^2^, conventional *R*-factors *R* are based on *F*, with *F* set to zero for negative *F*^2^. The threshold expression of *F*^2^ > σ(*F*^2^) is used only for calculating *R*-factors(gt) *etc*. and is not relevant to the choice of reflections for refinement. *R*-factors based on *F*^2^ are statistically about twice as large as those based on *F*, and *R*- factors based on ALL data will be even larger.

### Fractional atomic coordinates and isotropic or equivalent isotropic displacement parameters (Å^2^)

**Table:**

	*x*	*y*	*z*	*U*_iso_*/*U*_eq_	
C3	0.72085 (13)	0.4404 (3)	0.61785 (14)	0.0621 (7)	
C4	0.70766 (15)	0.5417 (3)	0.57753 (16)	0.0718 (8)	
H4	0.7341	0.6117	0.5874	0.086*	
C5	0.65605 (15)	0.5416 (2)	0.52285 (14)	0.0611 (7)	
H5	0.6470	0.6104	0.4957	0.073*	
C6	0.61833 (12)	0.43645 (18)	0.50978 (11)	0.0441 (5)	
C7	0.53305 (12)	0.30227 (16)	0.46674 (10)	0.0372 (5)	
C8	0.58105 (11)	0.23360 (16)	0.52297 (9)	0.0339 (4)	
C1	0.63164 (11)	0.33335 (17)	0.54984 (11)	0.0404 (5)	
C2	0.68261 (11)	0.3336 (2)	0.60484 (12)	0.0483 (5)	
H2	0.6913	0.2651	0.6323	0.058*	
C11	0.70709 (15)	0.2294 (2)	0.38484 (14)	0.0646 (7)	
H11A	0.7397	0.1972	0.3487	0.097*	
H11B	0.7348	0.2821	0.4158	0.097*	
H11C	0.6675	0.2736	0.3622	0.097*	
C10	0.67503 (11)	0.12915 (19)	0.42792 (11)	0.0430 (5)	
N2	0.69504 (10)	0.01800 (17)	0.41311 (10)	0.0512 (5)	
C12	0.61464 (11)	0.01774 (17)	0.50416 (10)	0.0369 (4)	
C9	0.62300 (11)	0.13389 (16)	0.48535 (10)	0.0352 (4)	
C14	0.53339 (10)	0.17653 (16)	0.58196 (9)	0.0328 (4)	
C13	0.53320 (11)	0.05668 (16)	0.59824 (9)	0.0342 (4)	
N3	0.57236 (9)	−0.02562 (14)	0.55869 (8)	0.0410 (4)	
H3	0.5702	−0.1014	0.5680	0.049*	
C18	0.49051 (12)	0.00573 (18)	0.66015 (10)	0.0419 (5)	
H18A	0.5191	−0.0597	0.6810	0.050*	
H18B	0.4440	−0.0276	0.6424	0.050*	
C17	0.47298 (12)	0.09738 (19)	0.71891 (10)	0.0430 (5)	
C16	0.43875 (12)	0.20721 (19)	0.68196 (11)	0.0450 (5)	
H16A	0.3899	0.1854	0.6638	0.054*	
H16B	0.4319	0.2702	0.7175	0.054*	
C15	0.48421 (11)	0.25641 (17)	0.62112 (10)	0.0365 (4)	
C20	0.54316 (14)	0.1327 (2)	0.75966 (12)	0.0585 (6)	
H20A	0.5308	0.1907	0.7960	0.088*	
H20B	0.5786	0.1669	0.7268	0.088*	
H20C	0.5644	0.0626	0.7819	0.088*	
C19	0.41680 (15)	0.0438 (2)	0.77122 (13)	0.0644 (7)	
H19A	0.4374	−0.0274	0.7927	0.097*	
H19B	0.3719	0.0235	0.7459	0.097*	
H19C	0.4057	0.1015	0.8081	0.097*	
N1	0.56218 (10)	0.41421 (14)	0.45939 (9)	0.0460 (4)	
H1	0.5478	0.4654	0.4276	0.055*	
O2	0.65528 (8)	−0.05644 (12)	0.46364 (7)	0.0470 (4)	
O1	0.48047 (8)	0.26181 (12)	0.43288 (7)	0.0459 (4)	
O3	0.47829 (9)	0.36227 (12)	0.60321 (8)	0.0500 (4)	
Cl1	0.78469 (4)	0.44523 (9)	0.68787 (4)	0.1018 (3)	

### Atomic displacement parameters (Å^2^)

**Table:**

	*U*^11^	*U*^22^	*U*^33^	*U*^12^	*U*^13^	*U*^23^
C3	0.0460 (14)	0.0766 (19)	0.0638 (15)	−0.0167 (13)	0.0158 (12)	−0.0339 (14)
C4	0.0681 (17)	0.0589 (17)	0.089 (2)	−0.0330 (14)	0.0337 (16)	−0.0327 (15)
C5	0.0736 (17)	0.0354 (12)	0.0744 (17)	−0.0175 (11)	0.0311 (15)	−0.0130 (11)
C6	0.0548 (13)	0.0296 (11)	0.0478 (11)	−0.0074 (9)	0.0184 (10)	−0.0068 (9)
C7	0.0561 (13)	0.0227 (10)	0.0327 (9)	0.0033 (9)	0.0095 (9)	0.0017 (8)
C8	0.0453 (11)	0.0244 (9)	0.0321 (9)	0.0000 (8)	0.0028 (8)	−0.0019 (7)
C1	0.0475 (12)	0.0326 (11)	0.0413 (10)	−0.0037 (9)	0.0102 (9)	−0.0094 (8)
C2	0.0436 (12)	0.0506 (13)	0.0506 (12)	−0.0015 (10)	0.0073 (10)	−0.0184 (10)
C11	0.0714 (17)	0.0578 (16)	0.0646 (15)	−0.0068 (13)	0.0226 (13)	−0.0022 (12)
C10	0.0437 (12)	0.0454 (13)	0.0398 (11)	0.0003 (10)	0.0003 (9)	−0.0071 (9)
N2	0.0558 (11)	0.0495 (12)	0.0484 (10)	0.0047 (9)	0.0043 (9)	−0.0093 (9)
C12	0.0457 (12)	0.0294 (10)	0.0357 (10)	0.0046 (9)	−0.0051 (9)	−0.0056 (8)
C9	0.0425 (11)	0.0297 (10)	0.0335 (9)	0.0007 (8)	−0.0018 (8)	−0.0038 (8)
C14	0.0451 (11)	0.0250 (9)	0.0285 (9)	−0.0002 (8)	−0.0009 (8)	0.0005 (7)
C13	0.0445 (11)	0.0278 (10)	0.0303 (9)	−0.0012 (8)	−0.0061 (8)	−0.0009 (7)
N3	0.0613 (11)	0.0222 (8)	0.0395 (9)	0.0039 (7)	−0.0019 (8)	0.0013 (7)
C18	0.0578 (13)	0.0302 (10)	0.0378 (10)	−0.0050 (9)	−0.0038 (9)	0.0073 (8)
C17	0.0569 (13)	0.0395 (11)	0.0327 (10)	−0.0024 (10)	0.0033 (9)	0.0049 (8)
C16	0.0541 (13)	0.0392 (12)	0.0418 (11)	0.0018 (10)	0.0077 (10)	0.0023 (9)
C15	0.0474 (11)	0.0284 (10)	0.0337 (9)	0.0002 (8)	−0.0013 (9)	−0.0006 (8)
C20	0.0742 (17)	0.0651 (16)	0.0361 (11)	−0.0030 (13)	−0.0064 (11)	−0.0025 (11)
C19	0.0755 (17)	0.0656 (16)	0.0520 (13)	−0.0035 (13)	0.0155 (13)	0.0179 (12)
N1	0.0712 (12)	0.0234 (8)	0.0433 (9)	−0.0020 (8)	0.0098 (9)	0.0063 (7)
O2	0.0580 (9)	0.0355 (8)	0.0474 (8)	0.0102 (7)	0.0002 (7)	−0.0086 (6)
O1	0.0630 (9)	0.0329 (8)	0.0418 (8)	−0.0007 (7)	−0.0082 (7)	0.0052 (6)
O3	0.0726 (10)	0.0265 (7)	0.0508 (9)	0.0072 (7)	0.0112 (8)	0.0040 (6)
Cl1	0.0629 (5)	0.1486 (8)	0.0938 (6)	−0.0320 (5)	−0.0028 (4)	−0.0515 (5)

### Geometric parameters (Å, º)

**Table:**

C3—C4	1.373 (4)	C12—C9	1.346 (3)
C3—C2	1.392 (3)	C12—N3	1.355 (2)
C3—Cl1	1.735 (3)	C14—C13	1.366 (2)
C4—C5	1.375 (4)	C14—C15	1.448 (3)
C4—H4	0.9300	C13—N3	1.368 (2)
C5—C6	1.372 (3)	C13—C18	1.494 (3)
C5—H5	0.9300	N3—H3	0.8600
C6—C1	1.387 (3)	C18—C17	1.527 (3)
C6—N1	1.397 (3)	C18—H18A	0.9700
C7—O1	1.220 (2)	C18—H18B	0.9700
C7—N1	1.356 (2)	C17—C20	1.520 (3)
C7—C8	1.555 (3)	C17—C19	1.522 (3)
C8—C9	1.511 (3)	C17—C16	1.529 (3)
C8—C1	1.517 (3)	C16—C15	1.498 (3)
C8—C14	1.528 (2)	C16—H16A	0.9700
C1—C2	1.372 (3)	C16—H16B	0.9700
C2—H2	0.9300	C15—O3	1.227 (2)
C11—C10	1.488 (3)	C20—H20A	0.9600
C11—H11A	0.9600	C20—H20B	0.9600
C11—H11B	0.9600	C20—H20C	0.9600
C11—H11C	0.9600	C19—H19A	0.9600
C10—N2	1.315 (3)	C19—H19B	0.9600
C10—C9	1.419 (3)	C19—H19C	0.9600
N2—O2	1.441 (2)	N1—H1	0.8600
C12—O2	1.333 (2)		
			
C4—C3—C2	121.3 (2)	C15—C14—C8	116.57 (15)
C4—C3—Cl1	119.9 (2)	C14—C13—N3	122.11 (17)
C2—C3—Cl1	118.8 (2)	C14—C13—C18	122.80 (17)
C3—C4—C5	121.3 (2)	N3—C13—C18	115.09 (16)
C3—C4—H4	119.4	C12—N3—C13	116.88 (15)
C5—C4—H4	119.4	C12—N3—H3	121.6
C6—C5—C4	117.6 (2)	C13—N3—H3	121.6
C6—C5—H5	121.2	C13—C18—C17	113.85 (16)
C4—C5—H5	121.2	C13—C18—H18A	108.8
C5—C6—C1	121.6 (2)	C17—C18—H18A	108.8
C5—C6—N1	128.7 (2)	C13—C18—H18B	108.8
C1—C6—N1	109.76 (17)	C17—C18—H18B	108.8
O1—C7—N1	125.72 (18)	H18A—C18—H18B	107.7
O1—C7—C8	126.47 (16)	C20—C17—C19	109.29 (18)
N1—C7—C8	107.75 (17)	C20—C17—C18	111.00 (18)
C9—C8—C1	113.00 (16)	C19—C17—C18	109.45 (18)
C9—C8—C14	107.84 (15)	C20—C17—C16	110.52 (18)
C1—C8—C14	113.66 (15)	C19—C17—C16	109.49 (18)
C9—C8—C7	108.91 (14)	C18—C17—C16	107.06 (16)
C1—C8—C7	101.19 (15)	C15—C16—C17	114.35 (17)
C14—C8—C7	112.14 (15)	C15—C16—H16A	108.7
C2—C1—C6	120.90 (19)	C17—C16—H16A	108.7
C2—C1—C8	130.20 (19)	C15—C16—H16B	108.7
C6—C1—C8	108.89 (18)	C17—C16—H16B	108.7
C1—C2—C3	117.3 (2)	H16A—C16—H16B	107.6
C1—C2—H2	121.3	O3—C15—C14	120.25 (17)
C3—C2—H2	121.3	O3—C15—C16	120.52 (18)
C10—C11—H11A	109.5	C14—C15—C16	119.19 (17)
C10—C11—H11B	109.5	C17—C20—H20A	109.5
H11A—C11—H11B	109.5	C17—C20—H20B	109.5
C10—C11—H11C	109.5	H20A—C20—H20B	109.5
H11A—C11—H11C	109.5	C17—C20—H20C	109.5
H11B—C11—H11C	109.5	H20A—C20—H20C	109.5
N2—C10—C9	111.83 (19)	H20B—C20—H20C	109.5
N2—C10—C11	119.01 (19)	C17—C19—H19A	109.5
C9—C10—C11	129.16 (19)	C17—C19—H19B	109.5
C10—N2—O2	105.51 (16)	H19A—C19—H19B	109.5
O2—C12—C9	112.65 (17)	C17—C19—H19C	109.5
O2—C12—N3	120.60 (17)	H19A—C19—H19C	109.5
C9—C12—N3	126.73 (17)	H19B—C19—H19C	109.5
C12—C9—C10	103.49 (17)	C7—N1—C6	111.84 (17)
C12—C9—C8	121.84 (17)	C7—N1—H1	124.1
C10—C9—C8	134.67 (17)	C6—N1—H1	124.1
C13—C14—C15	119.00 (17)	C12—O2—N2	106.51 (14)
C13—C14—C8	124.41 (16)		
			
C2—C3—C4—C5	0.1 (4)	C1—C8—C9—C10	−53.1 (3)
Cl1—C3—C4—C5	−178.35 (18)	C14—C8—C9—C10	−179.6 (2)
C3—C4—C5—C6	−0.2 (3)	C7—C8—C9—C10	58.5 (3)
C4—C5—C6—C1	−0.3 (3)	C9—C8—C14—C13	2.9 (2)
C4—C5—C6—N1	177.8 (2)	C1—C8—C14—C13	−123.2 (2)
O1—C7—C8—C9	65.4 (2)	C7—C8—C14—C13	122.75 (19)
N1—C7—C8—C9	−111.86 (17)	C9—C8—C14—C15	−175.48 (16)
O1—C7—C8—C1	−175.38 (18)	C1—C8—C14—C15	58.4 (2)
N1—C7—C8—C1	7.40 (18)	C7—C8—C14—C15	−55.6 (2)
O1—C7—C8—C14	−53.9 (2)	C15—C14—C13—N3	173.17 (17)
N1—C7—C8—C14	128.87 (16)	C8—C14—C13—N3	−5.1 (3)
C5—C6—C1—C2	0.9 (3)	C15—C14—C13—C18	−6.8 (3)
N1—C6—C1—C2	−177.55 (17)	C8—C14—C13—C18	174.89 (17)
C5—C6—C1—C8	179.47 (18)	O2—C12—N3—C13	179.11 (16)
N1—C6—C1—C8	1.1 (2)	C9—C12—N3—C13	1.0 (3)
C9—C8—C1—C2	−70.3 (2)	C14—C13—N3—C12	3.1 (3)
C14—C8—C1—C2	53.0 (3)	C18—C13—N3—C12	−176.94 (16)
C7—C8—C1—C2	173.4 (2)	C14—C13—C18—C17	−22.5 (3)
C9—C8—C1—C6	111.28 (18)	N3—C13—C18—C17	157.52 (17)
C14—C8—C1—C6	−125.39 (17)	C13—C18—C17—C20	−70.7 (2)
C7—C8—C1—C6	−5.00 (19)	C13—C18—C17—C19	168.61 (18)
C6—C1—C2—C3	−0.9 (3)	C13—C18—C17—C16	50.0 (2)
C8—C1—C2—C3	−179.21 (19)	C20—C17—C16—C15	68.5 (2)
C4—C3—C2—C1	0.5 (3)	C19—C17—C16—C15	−171.06 (18)
Cl1—C3—C2—C1	178.95 (15)	C18—C17—C16—C15	−52.5 (2)
C9—C10—N2—O2	−0.7 (2)	C13—C14—C15—O3	−173.34 (18)
C11—C10—N2—O2	179.71 (19)	C8—C14—C15—O3	5.1 (3)
O2—C12—C9—C10	−0.8 (2)	C13—C14—C15—C16	4.5 (3)
N3—C12—C9—C10	177.44 (18)	C8—C14—C15—C16	−177.07 (17)
O2—C12—C9—C8	178.75 (16)	C17—C16—C15—O3	−155.17 (19)
N3—C12—C9—C8	−3.0 (3)	C17—C16—C15—C14	27.0 (3)
N2—C10—C9—C12	1.0 (2)	O1—C7—N1—C6	175.33 (18)
C11—C10—C9—C12	−179.5 (2)	C8—C7—N1—C6	−7.4 (2)
N2—C10—C9—C8	−178.5 (2)	C5—C6—N1—C7	−174.1 (2)
C11—C10—C9—C8	1.0 (4)	C1—C6—N1—C7	4.2 (2)
C1—C8—C9—C12	127.5 (2)	C9—C12—O2—N2	0.4 (2)
C14—C8—C9—C12	1.0 (2)	N3—C12—O2—N2	−177.96 (16)
C7—C8—C9—C12	−120.92 (19)	C10—N2—O2—C12	0.2 (2)

### Hydrogen-bond geometry (Å, º)

**Table:**

*D*—H···*A*	*D*—H	H···*A*	*D*···*A*	*D*—H···*A*
N1—H1···O3^i^	0.86	2.05	2.837 (2)	151
N3—H3···O1^ii^	0.86	2.00	2.795 (2)	153

Symmetry codes: (i) −*x*+1, −*y*+1, −*z*+1; (ii) −*x*+1, −*y*, −*z*+1.
